# Risk Compensation Is Not Associated with Male Circumcision in Kisumu, Kenya: A Multi-Faceted Assessment of Men Enrolled in a Randomized Controlled Trial

**DOI:** 10.1371/journal.pone.0002443

**Published:** 2008-06-18

**Authors:** Christine L. Mattson, Richard T. Campbell, Robert C. Bailey, Kawango Agot, J. O. Ndinya-Achola, Stephen Moses

**Affiliations:** 1 School of Public Health, University of Illinois at Chicago, Chicago, Illinois, United States of America; 2 UNIM Project, Kisumu, Kenya; 3 Department of Medical Microbiology, University of Nairobi, Nairobi, Kenya; 4 Department of Medical Microbiology, University of Manitoba, Winnipeg, Canada; University of Cape Town, South Africa

## Abstract

**Background:**

Three randomized controlled trials (RCTs) have confirmed that male circumcision (MC) significantly reduces acquisition of HIV-1 infection among men. The objective of this study was to perform a comprehensive, prospective evaluation of risk compensation, comparing circumcised versus uncircumcised controls in a sample of RCT participants.

**Methods and Findings:**

Between March 2004 and September 2005, we systematically recruited men enrolled in a RCT of MC in Kenya. Detailed sexual histories were taken using a modified Timeline Followback approach at baseline, 6, and 12 months. Participants provided permission to obtain circumcision status and laboratory results from the RCT. We evaluated circumcised and uncircumcised men's sexual behavior using an 18-item risk propensity score and acquisition of incident infections of gonorrhea, chlamydia, and trichomoniasis. Of 1780 eligible RCT participants, 1319 enrolled (response rate = 74%). At the baseline RCT visit, men who enrolled in the sub-study reported the same sexual behaviors as men who did not. We found a significant reduction in sexual risk behavior among both circumcised and uncircumcised men from baseline to 6 (p<0.01) and 12 (p = 0.05) months post-enrollment. Longitudinal analyses indicated no statistically significant differences between sexual risk propensity scores or in incident infections of gonorrhea, chlamydia, and trichomoniasis between circumcised and uncircumcised men. These results are based on the most comprehensive analysis of risk compensation yet done.

**Conclusion:**

In the context of a RCT, circumcision did not result in increased HIV risk behavior. Continued monitoring and evaluation of risk compensation associated with circumcision is needed as evidence supporting its' efficacy is disseminated and MC is widely promoted for HIV prevention.

## Introduction

The results of three randomized controlled trials (RCTs) have demonstrated that male circumcision (MC) significantly reduces acquisition of heterosexually transmitted HIV-1 among men,[1]–[3] confirming observational evidence [4], [5] and adding plausibility to previous immunohistochemistry studies of the foreskin. [6], [7] All three trials were stopped prematurely by their respective Data and Safety Monitoring Boards when, at interim analyses, circumcision was found to be highly efficacious in reducing HIV incidence, indicating that it would be unethical to continue withholding circumcision from the control group. [8]–[10] The protective effect of male circumcision ranged from 51% to 60% in intent to treat analyses, and from 60% to 76% in as treated analyses. Despite these results, concern about how men perceive the protective effect of circumcision remains. [11] If circumcised men believe that circumcision confers substantial or complete protection against HIV infection, they may engage in increased risk behavior, commonly referred to as risk compensation or behavioral disinhibition. [12] Significant risk compensation could reduce the protective effect of circumcision and possibly result in increased rather than decreased incidence of HIV.

The empirical evidence regarding risk compensation in the context of male circumcision is inconsistent. Two cross-sectional studies have indicated that circumcised men engage in higher risk behaviors than uncircumcised men; however, given the observational nature of the studies, these results could reflect confounding. [13], [14] In contrast, a prospective cohort study conducted in Siaya and Bondo Districts, Kenya found that circumcised men did not have more extra-marital sex partners than uncircumcised men. [15] Data from the RCTs of MC did not show consistent evidence of risk compensation; however, minimal increases in some risky behaviors were noted. [1]–[3] In the Orange Farm study, the following five variables were evaluated at baseline and at 3, 12, and 21 months later: at least one sexual contact without a condom, being married or living as married, more than 1 non-spousal partner, at least one sexual partnership with only one sexual contact, and more than 5 sexual contacts. Only differences in mean number of sexual contacts were statistically significant between 4–12 months after circumcision (5.9 versus 5.0, p<0.001) and 13–21 months (7.5 versus 6.4, p = 0.0015). [1] Controlling for behavioral differences between study groups altered the protective effect against HIV acquisition minimally from 60% (95% CI 32%–76%) to 61% (95% CI 34%–77%). [1] All other differences were non-significant.

The Ugandan trial found no consistent evidence of behavioral disinhibition. [3] In this study, the following sexual behaviors were evaluated: condom use (defined as none, inconsistent, or consistent), number of sexual partners (0, 1, 2, 3+), any non-marital partners, alcohol use with sexual intercourse (none versus any), and transactional sex (exchanging money or gifts for sex). There was no difference in the proportion of circumcised and uncircumcised men reporting consistent condom use at the 6, 12 and 24 month follow-up visit (p = 0.11, p = 0.6, p = 1.0, respectively). Inconsistent condom use was higher in the circumcised group (p = 0.0004) at the 6 month follow-up visit, but men in the control group were more likely to report no condom use compared to circumcised men (p = 0.0004). [3] At the 12 and 24 month follow-up visits, number of sexual partners, non-marital relationships, and condom use were similar in both groups. Alcohol use with sexual intercourse was the same at enrollment, but higher among uncircumcised men at the 6 month (p = 0.001), 12 month (p = 0.06), and 24 month follow-up visit (p = 0.02). Transactional sex did not differ between groups.

In the Kenya trial, the following sexual behavior variables were evaluated: unprotected intercourse with any partner in the previous 6 months, last sexual relations with a casual partner, sexual abstinence in the last 6 months, consistent condom use in the previous 6 months, and 2 or more partners in the previous 6 months. There was a reduction in risk behavior among both circumcised and uncircumcised men from baseline to follow-up visits, except in the proportion of men reporting 2 or more sex partners, which progressively declined in the control group throughout the duration of follow-up, but declined and then stabilized after the 6 month follow-up visit in circumcised men. [2] At the 24 month follow-up visit, the proportion of circumcised men reporting unprotected sexual intercourse in the previous six months was greater than the control group (p = 0.03), and fewer circumcised men reported consistent condom use versus controls (p = 0.03), which could suggest that men's behavior became refractory to prevention messages. [2] It is notable that risk behaviors declined for both circumcised and uncircumcised men from baseline to 24 months of follow-up, which the authors attribute to the risk reduction counseling that participants received. [2] It was suggested that circumcised men not only did not increase their sexual risk behaviors after circumcision, they reduced their risk, but there were even greater reductions from baseline to month 24 in the control group. [2]

At most, 5 behaviors were evaluated in the context of the trials and the behaviors chosen to indicate sexual risk varied across studies. Measuring condom use and sex with multiple partners is conventional in studies of risk compensation, but how those variables are defined and what other behaviors are chosen to represent risk are not consistent. This is due, in part, to the lack of a consensus definition of “sexual risk,” as well as to the statistical challenges inherent in analyzing multiple, highly correlated behavioral variables. [15] It is possible that information obtained from relatively brief questionnaires lacks sufficient breadth to comprehensively address the issue of risk compensation. This study was specifically designed to measure and analyze a comprehensive set of sexual behaviors by applying a behavioral risk propensity scale developed to assess risk compensation in circumcised versus uncircumcised men participating in the circumcision trial in Kenya.

## Methods

### Design and Data Collection

The Kenyan RCT of MC enrolled men who were sexually active within the last 12 months, were HIV negative, uncircumcised at baseline, aged 18–24 years, and resident in Kisumu District. The men received HIV testing and counseling on risk reduction strategies following Kenyan National Guidelines at the following RCT visits: 1, 3, 6, and 12 month. All men who enrolled in the RCT between March 2004 and September 2005 were systematically invited to participate in the current study, which took place at a separate facility approximately 100 meters from the trial study site. The men received HIV testing and counseling on risk reduction strategies following Kenyan National Guidelines at the following RCT visits: 1, 3, 6, and 12 month. All men were informed that although there was some evidence of an association between circumcision and reduced HIV acquisition, the evidence was not conclusive. Participants provided written informed consent to undergo separate interviews for this study at baseline and at 6 and 12 month follow-up visits, and allowed us to obtain data from the RCT. The questionnaires and consent documents were developed in English and translated into Dholuo and Kiswahili (the predominant local languages) independently by two indigenous speakers, and discrepancies were resolved with assistance from a third individual. Interviews were conducted by male interviewers fluent in all three languages. Men were offered 150 Kenyan shillings (approximately $2.00 USD) for each scheduled visit to cover transport and loss of income. The research protocol was approved by the Kenyatta National Hospital Ethics and Research committee, the University of Illinois at Chicago's Institutional Review Board #3, and the University of Manitoba Biomedical Research Ethics Board.

To obtain sexual histories, we adapted the well-validated Timeline Followback approach [16] to collect information about every sexual relationship in the last 6 months for up to 12 partners at baseline (within 10 days of being randomized in the trial), and at 6 and 12 month follow up visits (plus or minus 3 months). Interviewers obtained the following information for each partner: age, gender, type of partner, dates of the relationship, length of time knowing the partner prior to sex, approximate number of sexual encounters, sexual practices (vaginal, oral, anal), exchange of sex for money or gifts, condom use (ever used a condom with the partner, used at the first encounter, at last encounter, and at every encounter), and clients' perception of their partners (e.g. if partners had other partners concurrently, engaged in transactional sex, or were thought to be HIV positive). Men were identified as having a concurrent partnership if the start and end dates of any two partners overlapped by at least one month. Men were also asked whether or not they thought circumcision reduced the risk of HIV. *A priori* power analyses indicated that 500 circumcised and 500 uncircumcised men would provide at least 80% power to detect an absolute difference of 7% among the proportion of men reporting unprotected sex with 2 or more partners between baseline and the 6 or 12 month follow-up visits with a base rate of .45 (paired proportion) among uncircumcised men with two-sided type 1 error of 0.05.

Using item response theory [17], [18], we developed an 18-item behavioral risk propensity scale to capture specific behaviors and practices that men reportedly engaged in with each partner discussed in their sexual histories (for up to 12 partners). Items were included in the scale if previous epidemiologic research demonstrated they were risk factors for HIV [19] and there was reason to believe the behavior may be affected by circumcision (e.g. condom use, multiple sex partners, transactional sex, etc). Count variables were created to summarize behaviors across 0–12 partnerships. Using differential item function analyses, we demonstrated that the scale performed consistently at all three time points, and whether interviews were conducted in English or not (Dholuo or Kiswahili). The resulting logit risk scores were transformed so that the lowest logit score (−4.62) was equal to zero. Transformed scores ranging from 0 to 9.24 were computed at baseline, 6 and 12 month follow-up visits. The scale demonstrated very good reliability (Cronbach's alpha of 0.87) and, based on assessments of monotonicity; it resulted in a unidimensional continuum to represent sexual risk propensity (e.g. sexual behavior).

The scale's construct validity (i.e. whether it does, indeed, measure risky sexual behavior) was established by demonstrating that men's scores on the risk scale were associated with the presence of an incident sexually transmitted infection (STI) of *Neisseria gonorrhoeae*, *Chlamydia trachomatis*, and *Trichomonas vaginalis* or incident HIV (*in press*). At both follow-up visits men diagnosed with an incident STI had higher risk scores than uninfected men, but the difference was only statistically significant at the 6 month follow-up visit (Wilcoxon two-sided probability p = 0.01), presumably due to the small number of respondents with STIs at the 12 month follow-up visit (n = 24). At the 6 and 12 month follow-up visits, 8 and 4 men respectively were diagnosed with incident HIV infection. Similar to the STI analyses, men who seroconverted throughout the study had higher risk scores than those who did not. The non-parametric Savage Two-Sample Test with one-sided probability was borderline at 6 months (p = 0.07) and statistically significant at 12 months (p = 0.01).

To corroborate self-reported risk behavior, we independently evaluated incident STIs as a secondary outcome variable. Because these infections occurred at a relatively high rate in our study population, [20], [21] and are not generally considered to be associated with male circumcision, they provide ideal biologic markers of sexual risk behavior. [22], [23] Also, since men received treatment for their infections, it was possible to distinguish prevalent from incident infections. [24] We used the following diagnostic criteria to identify infections: *Neisseria gonorrhoeae* and *Chlamydia trachomatis*: by polymerase chain reaction assay (AMPLICOR® CT/NG Test, Roche Diagnostics, Montreal Canada) and *Trichomonas vaginalis*: by culture (InPouch™ TV test, Biomed Diagnostics, Oregon, United States). Infections identified at baseline were considered prevalent. Since men identified with an STI at baseline were treated, infections subsequent to baseline were considered incident.

### Statistical Analyses

Data were entered into SPSS Version 10.0 (SPSS Inc., Chicago, IL). Approximately 30% of interviews were entered in duplicate to evaluate accuracy. The error rate was less than 1%. Further data management, descriptive analyses, and multivariable modeling were performed in SAS Version 8.2 (SAS Institute Inc., Cary, North Carolina, USA).

To compare men who enrolled in the sub-study to those who did not and to compare circumcised to uncircumcised men at baseline, we used Pearson Chi-Square tests for categorical variables, independent t-tests for normally distributed continuous variables and Wilcoxon's Two Sample Z Test for continuous variables that were not normally distributed. To evaluate whether circumcision altered men's sexual risk behavior, we used random effects regression models to deal with statistical dependence resulting from repeated observations. [25]

The primary outcome was the 18-item risk score, which had a “semi-continuous” or “mixed” distribution ranging from 0 to 9. [26]–[28] Men who had not engaged in any sexual activity in the past 6 months had scores of 0, but men who were sexually active in the 6 months prior to the interview had scores ranging from >0–9. We implemented a two-part random effects regression model to accommodate the “semi-continuous” distribution. [27] The first equation in the model evaluated whether or not men engaged in any activity in the last 6 months as a binary yes/no outcome (modeled as logistic) and the second equation modeled the positive sexual risk score as a continuous outcome (modeled as lognormal to account for the skewed distribution). The covariates were interpreted as if the two parts of the model were fit separately but the random effects in the two equations were allowed to correlate. Population-averaged rather than subject-specific odds ratios are reported. [25] The model was estimated using SAS Proc Mixed, implemented via a set of SAS macros developed by Tooze and Grunwald. [24], [26]

The independent variables of interest were: circumcision group (men randomized to receive circumcision versus those randomized to remain uncircumcised); time (indicator variables for 6 month and 12 month follow-up); and the interaction between circumcision group and time. Additional covariates included age, marital status, education, income and the belief that circumcision reduces risk of acquiring HIV.

The secondary outcome variable included information on whether or not men were diagnosed with an incident infection of *Neisseria gonorrhoeae*, *Chlamydia trachomatis*, or *Trichomonas vaginalis* within 3 months before or after the behavioral interview at the 6^th^ and 12 month follow-up visit. Any incident STIs versus none were modeled using dichotomous random effects models using Proc NLMIXED. [29]

## Results

Between March 2004 and September 2005, 1780 men were enrolled in the RCT and therefore met eligibility criteria for this study. Of these, 1319 chose to participate in this study, yielding an overall response rate of 74%, which surpassed our target enrollment of 1000. Men who joined this sub-study were slightly more likely to have been randomized to the control (53%) versus circumcision (47%) arm (p<0.001), were younger (46% vs. 41% p = 0.03), more likely to have completed secondary school (58% vs. 52%, p = 0.03), and more likely to be unemployed (67% vs. 60%, p = 0.02) than those who did not enroll. There were no significant differences between the median number of lifetime sex partners (Wilcoxon Two Sample Z Test = 0.01, p = 0.95), number of sex partners in the last 6 months (χ^2^ = 0.53, p = 0.77), or occurrence of a prevalent sexually transmitted infection (χ^2^ = 0.17, p = 0.68) at baseline between the men who enrolled in this study versus those who did not.

Ten men with missing data on key outcome variables were eliminated from all subsequent analyses. The final sample included 1309 men, of whom 620 (47%) had been randomized to undergo circumcision and 689 (53%) were controls in the parent RCT. Table 1 compares the baseline characteristics of the enrolled circumcised and uncircumcised men. In general, the groups were similar, except that uncircumcised men were more likely to be unemployed (χ^2^ = 16.1, p = 0.01) and circumcised men were more likely to be diagnosed with a sexually transmitted infection (χ^2^ = 5.6, p = 0.02). In the main RCT, 11 men who were randomized to the control group became circumcised. Three of these men enrolled in this study. Of 57 men in the RCT who were randomized to receive circumcision never received the surgery, sixteen of these enrolled in this study. To investigate the effect of these crossovers, we performed an “as treated” analysis, which yielded the same conclusions as the “intent to treat” analysis, which we report here.

**Table 1:** Baseline Comparability of Men in Circumcised and Uncircumcised Group (n = 1309)*

Variable	Circumcised		Uncircumcised		p-value
	n = 620		n = 689		
	n	%	N	%	
Age					
18–20	342	55	366	53	0.46
21–24	278	45	323	47	
Language of Interview**					
English	355	57	418	61	0.32
DhoLuo	256	41	265	38	
Kiswahili	9	1	6	1	
Education**					
Primary (0–8)	116	19	129	19	0.10
Secondary (9–12)	353	57	391	57	
Post-Secondary (13 or more)	151	24	169	25	
Occupation					
Professional/Managerial	105	17	137	20	0.01
Service Worker/Casual worker	102	16	110	16	
Farmer/fisherman	83	13	64	9	
Student	122	20	139	19	
Other	64	10	46	7	
None	144	23	203	29	
Income					
2000 ksh/month or less	360	58	425	62	0.17
More than 2000 ksh/month	261	42	264	38	
Marital Status					
Married/cohabitating	42	7	50	7	0.73
Single	578	93	639	93	
Age at Sexual Debut					
<15	274	44	282	41	0.23
> = 15	346	56	407	59	
Number of Sex Partners last 6 mo					
None	46	7	50	7	0.89
One	259	42	277	40	
Two	159	26	176	26	
Three or more	156	25	186	27	
Diagnosed with a STI at baseline					
Yes	65	10	47	7	0.02
No	555	90	642	93	
Lifetime Sex Partners	Median = 5.0		Median = 5.0		0.72
	IQR = 1–8		IQR = 1–9		

* 16 men in “circumcised” group did not actually receive circumcisions and 3 men in the “uncircumcised” group received circumcisions.

** Percentages do not add up to 100 because of rounding.

Of the 1309 men enrolled, 1001 (76%) returned for the 6 month follow-up and 1007 (77%) returned at 12 months. There was no differential loss to follow-up between study groups (χ^2^ = 0.02., p = 0.90). Men who were interviewed at all three time points (n = 873) had slightly higher sexual risk propensity scores ( = 3.4) than men interviewed at one ( = 3.0) or two ( = 3.3) time points indicating that men with less risky sexual behavior were more likely to be lost to follow-up than men with high risk behavior (Wilcoxon Two Sample Z Test with a continuity correction of .5 = −1.93, p = 0.054).

Table 2 shows the 18 risk scale items, the STI data, and the belief that circumcision reduces the risk of HIV by group and time. There were no statistically significant differences between the proportion of circumcised and uncircumcised men who engaged in any of the 18 behaviors at any time point. Moreover, both circumcised and uncircumcised men reported lower numbers of total sex partners and lower numbers of partners with whom they did not always use a condom at follow-up visits than they did at baseline. Of note, circumcised men were no more likely to report the belief that circumcision reduces the risk of acquiring HIV than uncircumcised men at any time point. At baseline, 57% of circumcised and 56% of uncircumcised men reported that they thought circumcision reduces the risk of HIV. However by the 12 month follow-up visit, endorsement of this belief rose to 75% of circumcised and 76% of uncircumcised men.

**Table 2:** Sexual Risk Scale Items for Circumcised and Uncircumcised Men at Baseline, 6 and 12 Month Follow-up Visits*

Variable	Baseline		6 M Follow-up		12 M Follow-up	
	n = 1309		n = 1001		n = 1007	
	Circ	Uncirc	Circ	Uncirc	Circ	Uncirc
Total number of sex partners:						
2 or more	315 (51)	363 (53)	185 (39)	200 (38)	177 (37)	206 (39)
1	259 (42)	277 (40)	216 (46)	236 (44)	222 (47)	235 (44)
0	46 (7)	49 (7)	69 (15)	95 (18)	75 (16)	92 (17)
Had unprotected sex with more than 1 partner:						
Yes	172 (28)	190 (28)	80 (17)	73 (14)	64 (14)	64 (12)
No	448 (72)	499 (72)	390 (83)	458 (86)	410 (86)	469 (88)
Had unprotected sex with more than 1 “regular” partner:						
Yes	114 (18)	117 (17)	35 (7)	36 (7)	33 (7)	32 (6)
No	506 (82)	572 (83)	435 (93)	495 (93)	441 (93)	501 (94)
Had unprotected sex with more than 1 “casual” partner:						
Yes	38 (6)	48 (7)	22 (5)	18 (3)	10 (2)	21 (4)
No	582 (94)	641 (93)	448 (93)	513 (97)	464 (98)	512 (96)
Total number of unprotected partners (e.g. partners with whom a condom was not always worn):						
2 or more	172 (28)	190 (28)	80 (17)	73 (14)	64 (14)	64 (12)
1	275 (44)	292 (42)	181 (39)	197 (37)	195 (41)	205 (38)
0	173 (28)	207 (30)	209 (44)	261 (49)	215 (45)	264 (50)
Had a concurrent partnership:						
Yes	284 (46)	329 (48)	154 (33)	164 (31)	78 (16)	81 (15)
No	336 (54)	360 (52)	316 (67)	367 (69)	396 (84)	450 (85)
Had sex while a partner was menstruating:						
Yes	90 (15)	96 (14)	58 (12)	57 (11)	55 (12)	55 (10)
No	530 (85)	593 (86)	412 (88)	474 (89)	419 (88)	478 (90)
Had sex with a partner after knowing her < = day:						
Yes	106 (17)	119 (17)	58 (12)	57 (11)	43 (9)	59 (11)
No	516 (83)	570 (83)	412 (88)	474 (89)	431 (91)	474 (89)
Had unprotected sex after knowing a partner < = day:						
Yes	31 (5)	44 (6)	17 (4)	16 (3)	9 (2)	12 (2)
No	589 (95)	645 (94)	453 (96)	515 (97)	465 (98)	522 (98)
Had sex with a commercial sex worker:						
Yes	37 (6)	30 (4)	16 (3)	14 (3)	11 (2)	15 (3)
No	583 (94)	659 (96)	454 (97)	517 (97)	463 (98)	518 (97)
Had unprotected sex with a commercial sex worker:						
Yes	8 (1)	11 (2)	2 (<1)	3 (1)	1 (<1)	2 (<1)
No	612 (99)	678 (98)	468 (99)	528 (99)	473 (99)	531 (99)
Ever exchange money or gifts for sex with a partner not reported to be a commercial sex worker:						
Yes	113 (18)	116 (17)	47 (10)	46 (9)	35 (7)	44 (8)
No	507 (82)	579 (83)	423 (90)	485 (91)	439 (93)	489 (92)
Always exchange money or gifts for sex with a partner not reported to be a commercial sex worker:						
Yes	13 (2)	18 (3)	5 (1)	4 (1)	3 (1)	7 (1)
No	607 (87)	671 (97)	465 (99)	527 (99)	471 (99)	526 (99)
Believed that a partner had any other sexual partners at the time of the relationship:						
Yes	272 (44)	311 (45)	151 (32)	163 (31)	130 (27)	141 (26)
No	348 (56)	378 (55)	319 (68)	368 (69)	344 (73)	392 (74)
Believed that a partner had other “regular” sexual partners at the time of the relationship:						
Yes	221 (45)	274 (49)	126 (45)	131 (44)	106 (38)	125 (40)
No	273 (55)	288 (51)	153 (55)	168 (56)	176 (62)	185 (60)
Believed that a partner had other “casual” sexual partners at the time of the relationship:						
Yes	231 (49)	257 (47)	130 (47)	140 (47)	118 (43)	135 (43)
No	245 (51)	284 (53)	146 (53)	159 (53)	158 (57)	178 (57)
Believed that a partner had sex with other partners for money or gifts at the time of the relationship:						
Yes	136 (29)	153 (29)	70 (26)	64 (22)	41 (16)	54 (19)
No	327 (71)	369 (71)	202 (74)	230 (78)	216 (84)	233 (81)
Believed that a partner had HIV/AIDS:						
Yes	17 (3)	11 (2)	10 (2)	6 (1)	3 (1)	8 (2)
No	603 (97)	678 (98)	460 (98)	525 (99)	471 (99)	525 (98)
Believed Circumcision reduces risk of acquiring HIV*						
Yes	356 (57)	387 (56)	319 (68)	373 (70)	357 (75)	405 (76)
No	265 (43)	302 (44)	152 (32)	158 (30)	118 (25)	129 (24)
Laboratory Diagnosed infection of gonorrhea, chlamydia, or trichomoniasis*						
Yes	65 (10)	47 (7)	27 (6)	17 (3)	10 (2)	14 (3)
No	555 (90)	642 (93)	443 (94)	514 (97)	464 (98)	518 (97)

* These variables are not included in the 18-item scale.

Both groups were diagnosed with fewer incident sexually transmitted infections at the 12 month follow-up visit compared to the 6 month visit. Because infections at baseline were considered prevalent and not incident, a direct comparison cannot be made from baseline to follow-up visits. However, when looking at the proportion of men diagnosed with an STI, circumcised men were more likely than uncircumcised men to be diagnosed with a prevalent infection at baseline (OR = 1.6, p = 0.02) and an incident infection at the 6 month follow-up visit (OR = 1.8, p = 0.05). At the 12 month follow-up visit, there were no significant differences between the proportions of circumcised and uncircumcised men diagnosed with incident STIs.

**Figure 1:**
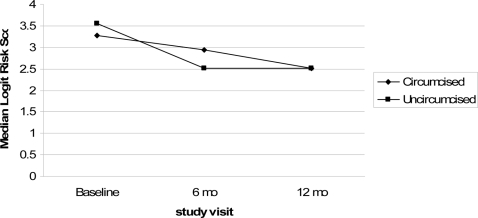
Median Risk Scores for Circumcised and Uncircumcised Men at Baseline, 6 and 12 month follow-up visits.

Median risk scores declined for all men (circumcised and uncircumcised) (Figure 1). Declining risk is confirmed by the two-part random effects regression models (Table 3). In the crude model evaluating circumcision and time, at the 6 month follow-up visit men were 57% less likely to engage in any sexual activity (OR = 0.43, 95% CI 0.34–0.54), and among men who had sex, there was a 12% decrease (expβ = 0.88, 95% CI 0.86–0.90) in risk scores compared to the baseline visit. Similarly, at the 12 month follow-up visit, men were 59% less likely to engage in any sex (OR = 0.41, 95% CI 0.33–0.51) and, of those who did, there was a 16% decrease in risk scores (expβ = 0.84, 95% CI 0.81–0.86) compared to baseline. There was no statistically significant difference in the risk scores of circumcised and uncircumcised men (OR = 1.09, 95% CI 0.86–1.38 for logistic and expβ = 1.02, 95% CI 0.99–1.01 for lognormal). Similarly, as shown in table 3, the interaction terms for circumcision group and time were not significant at the 6 month follow-up visit (OR = 1.27, 95% CI 0.83–1.95 for logistic and expβ = 1.01, 95% CI 0.96–1.06 for lognormal) or the 12 month follow-up visit (OR = 1.21, 95% CI 0.79–1.86 for logistic and expβ = 1.03, 95% CI 0.98–1.07 for lognormal). Thus, there was no evidence of differential risk according to circumcision group. Because it was not statistically significant, the interaction terms for circumcision status and visit were not included in the adjusted model. The random effects variance in both parts of the model was significant (p<0.01 for both) demonstrating that there was heterogeneity among men with respect to engaging in any sexual activity and in risk scores.

**Table 3:** Results of the Two-Part Random Effects Regression Modeling the Sexual Risk Score, Circumcision Status, and Time With and Without Covariates

	Crude Model Circumcision and Time		Crude Model with Group by Time Interaction		Adjusted Model	
	Sexually Active (y/n) Logistic	Risk Scores >0 Lognormal	Sexually Active (y/n) Logistic	Risk Scores >0 Lognormal	Sexually Active (y/n) Logistic	Risk Scores >0 Lognormal
	Expβ (95 % CI)	Expβ (95 % CI)	Expβ (95 % CI)	Expβ (95 % CI)	Expβ (95 % CI)	Expβ (95 % CI)
Circumcised vs. uncircumcised	1.09 (0.86–1.38)	1.02 (0.99–1.01)	0.92 (0.64–1.33)	1.01 (0.97–1.04)	1.07 (0.85–1.36)	1.01 (0.98–1.03)
6 month visit vs. baseline	0.43 (0.34–0.54)	0.88 (0.86–0.90)	0.39 (0.29–0.52)	0.88 (0.85–0.91)	0.40 (0.32–0.51)	0.90 (0.87–0.92)
12 month visit vs. baseline	0.41 (0.33–0.51)	0.84 (0.81–0.86)	0.37 (0.28–0.51)	0.84 (0.81–0.86)	0.37 (0.29–0.47)	0.86 (0.84–0.89)
Circumcision and 6 month visit			1.27 (0.83–1.95)	1.01 (0.96–1.06)		
Circumcision and 12 month visit			1.21 (0.79–1.86)	1.03 (0.98–1.07)		
Age (continuous)					1.00 (0.93–1.08)	1.00 (0.99–1.00)
Married/cohabitating vs. single					10.29 (4.19–25.5)	1.02 (0.98–1.06)
Primary school or less vs. more					1.12 (0.81–1.55)	1.03 (1.00–1.06)
<2000 vs. > = 2000					0.69 (0.53–0.89)	0.96 (0.94–0.98)
Believed circumcision reduces risk of HIV					1.09 (0.87–1.36)	1.00 (0.97–1.02)

In the logistic part of the model, exponentiated betas represent population averaged odds ratios.

In the lognormal portion of the model represent, exponentiated betas represent % change in Y (risk score), per change in unit X.

In the adjusted model, the following covariates were adjusted for: age, marital status, education, income, and belief that circumcision reduces the risk of acquiring HIV.

After adjusting for age, marital status, education, income, and the belief that circumcision status reduces the risk of acquiring HIV, there was still a significant decline in risk scores at the 6 and 12 month follow-up visits compared to baseline. Circumcision group remained non-significant. Men who were married or cohabitating with a woman were more likely to engage in any sexual activity (OR = 10.29, 95% CI 4.19–25.5) and had slightly higher mean risk scores (expβ = 1.02, 95% CI 0.98–1.06) than single men. Similarly, men who earned less than 2000 ksh/month (approximately $27 US) were less likely to engage in any sexual activity (OR = 0.69, 95% CI 0.53–0.89) and had lower mean risk scores (expβ = 0.96, 95% CI 0.94–0.98). Of note, although the proportion of men reporting the belief that circumcision reduces the risk of acquiring HIV increased from baseline (57%) to the 12 month follow-up visit (76%), this belief was not associated with engaging in any sexual activity (OR 1.09, 95% CI 0.87–1.36) or in higher risk scores (expβ = 1.00, 95% CI 0.97–1.02).

Table 4 presents the results of the crude model with circumcision and time, the model including the interaction between circumcision and time, and the adjusted random effect regression models comparing incident STIs at the 12 month visit compared to the 6 month visit. Like risk scores, circumcision was not associated with a statistically significant increase in STIs in the crude (OR 1.28, 95% CI 0.82–2.00) or adjusted models (OR 1.25, 95% CI 0.79–2.00). Fewer men were diagnosed with incident STIs at the 12 month visit compared to the 6 month visit (adjusted OR 0.55, 95% CI 0.34–0.88). The interaction between circumcision status and time indicated that circumcised men were slightly less likely to be diagnosed with an STI at the 12 month visit than their uncircumcised counterparts, but the term was not statistically significant (OR = 0.49, 95% CI 0.21–1.21).

**Table 4:** Results of the Dichotomous Random Effects Regression Models for Incident Infections of Gonorrhea, Chlamydia, or Trichomoniasis, Circumcision Status, and Time With and Without Covariates

	Crude Model Group by Time		Crude Model with Group by Time Interaction		Adjusted Model*	
	OR† (p-value)	95% CI	OR† (p-value)	95% CI	OR† (p-value)	95% CI
Circumcised vs. uncircumcised	1.28 (0.28)	0.82–2.00	1.65 (0.08)	0.94–2.88	1.25 (0.34)	0.79–2.00
12 compared to 6 month visit	0.57 (0.01)	0.36–0.89	0.82 (0.52)	0.44–1.53	0.55 (0.01)	0.34–0.88
Circumcision by change from 6 to 12 month visit			0.49 (0.12)	0.20–1.21		
Prevalent STI at baseline visit					3.07 (0.01)	1.65–5.71
Age (continuous)					0.99 (0.85)	0.85–1.14
Married/cohabitating vs. single					1.02 (0.96)	0.52–1.97
Primary school or less vs. more					1.03 (0.92)	0.57–1.86
<2000 ksh/month vs. > = 2000 ksh/month					0.40 (0.01)	0.24–0.66
Believed circumcision reduces risk of HIV					1.09 (0.73)	0.65–1.84
Random Effects σ^2^	1.09		1.02		0.80	

* The following co-variates were adjusted for: age, marital status, education, and income.

† All odds ratios are population averaged, subject-specific not shown.

## Discussion

We used two different measures of sexual risk to evaluate risk compensation associated with male circumcision: an index based on 18 sexual behaviors and laboratory diagnosed STIs. We found no evidence to suggest that circumcised men engaged in increased risk behavior after the procedure. To the contrary, both circumcised and uncircumcised men significantly reduced their HIV risk behavior from baseline to the 6 and 12 month follow-up visits. This decline was evident when evaluating the overall risk score across time and by comparing the individual 18 behaviors at a given time point. There was also a decline in the number of incident infections of gonorrhea, chlamydia and trichomoniasis from the 6 month visit to the 12 month visit. The overall consistency of the self-reported sexual behavior data with the biologic indicator (STI) likely indicates a low degree of misreported sexual behavior.

Circumcised men were slightly more likely to be diagnosed with incident STIs than uncircumcised men at the 6 month visit (6% vs. 3%, p = 0.05). However, it is important to note that circumcised men were also more likely to be diagnosed with an STI at baseline (10% vs. 7%, p = 0.02). Even though all men received treatment and referrals for their partners to receive treatment, it is possible that sex partners did not receive treatment and potentially re-infected the men. Ideally, we would have compared incident infections at all three time points, but it was not possible to disentangle prevalent from incident infections at the baseline visit. Given that limitation, longitudinal analyses indicated that incident STIs declined from the 6 month to the 12 month visit and there was no interaction between circumcision and time.

In general, our findings are consistent with most empirical studies of behavioral disinhibition in the context of male circumcision. [2], [3], [15] This sub-study included approximately half the participants enrolled in the RCT, but used a different methodology that was specifically designed to assess a comprehensive combination of risk behaviors. Instead of focusing on 5 sexual risk behaviors as was done in the RCT, we evaluated 18 risk behaviors/sexual practices and evaluated the occurrence of incident STIs. Similar to the conclusions drawn from the trials in Kenya and Uganda and a prospective study in Siaya and Bondo districts in Kenya, we found no compelling evidence of increased risk behavior among circumcised men. [2], [3], [15] As was the case in the overall results from the Kenyan trial, we detected an equal reduction in risky sexual practices among both circumcised and uncircumcised men. [2] That the results from the RCT and from this study were consistent strengthens the conclusions of both studies. These are important results in the face of reluctance on the part of some in the international health community to endorse male circumcision. [30] The consistency and strength of the results presented in this study, the RCT, and in previous studies, [1]–[3], [15] provide evidence that risk compensation is likely to be minimal or absent among circumcised men and, therefore, it should not *a priori* be considered a barrier to the promotion of male circumcision for HIV and STI prevention.

There were several limitations to this study. All the participants were from a narrow age range (18–24 years) and were enrolled in a RCT which accepted only healthy, sexually active HIV-uninfected individuals. When MC services are made widely available, it is likely that men and boys from a wide spectrum of ages will access the services and, in the absence of HIV testing, some may be HIV infected prior to circumcision. Of those eligible for this study, 74% enrolled. We found no differences in sexual behavior between those enrolled and those not enrolled but eligible. Nevertheless, there could have been differences that we were unable to measure. Further, because this was an observational study, we were unable to control for unknown confounders. In addition, the participants were followed for only twelve months. Although logically one might think that risk compensation would occur soon after wound healing in the circumcised men, it is possible that circumcised men become less sexually inhibited after they have been in their new status for more than one year. Finally, as with all studies that rely on participants' self-reports, misreporting of behaviors was possible. Study interviewers were carefully trained and the questionnaire using the Timeline Followback approach had many means of checking for inconsistencies. We also used biologic outcomes of risk (STIs), and these were consistent with the results from the self-reported behaviors.

Participation in the main RCT and in this study entailed repeated HIV testing and individually-tailored risk reduction counseling at the baseline, 6 month, and 12 month study visits, and participants were informed that the evidence for MC having a protective effect against HIV acquisition was inconclusive. Conditions under which MC is provided widely are likely to be different. Consequently, despite there now being consistent findings from five studies that risk compensation is essentially absent after circumcision, [1]–[3], [15] it will be necessary to further evaluate the possibility that men increase their HIV risk behavior after circumcision is offered in more naturalistic public health and medical settings. Alternatively and preferably, until further evidence becomes available, as MC services are introduced and promoted, the HIV prevention community should ensure that MC services are integrated with a full package of HIV prevention measures including HIV testing, STI diagnosis and treatment, condom provision, and risk reduction counseling. The results of this study suggest that, under such conditions, HIV risk behaviors after circumcision are unlikely to increase. Indeed, they may well decline.
